# The Work Behind the Work: Logistical–Administrative Workload, Health Technology Satisfaction and Missed Nursing Care Among Belgian Hospital Nurses

**DOI:** 10.1155/jonm/4981208

**Published:** 2026-08-30

**Authors:** Meryl Paquay, Giordano Bellaccomo, Jérôme E. Dauvergne, Arnaud Bruyneel

**Affiliations:** ^1^ Public Health Department, Faculty of Medicine, University of Liege, Liege, Belgium, ulg.ac.be; ^2^ Emergency Department, Liège University Hospital, Liège, Belgium, chuliege.be; ^3^ Health Economics, Hospital Management and Nursing Research Department, School of Public Health, Université Libre de Bruxelles, Brussels, Belgium, ulb.ac.be; ^4^ Department of Anaesthesiology and Critical Care, Laënnec Hospital, CHU Nantes, Nantes Université, Nantes F-44000, France, chu-nantes.fr

**Keywords:** missed care, nurses, nursing staff, patient safety, satisfaction, technology, workload

## Abstract

**Background:**

Hospital nurses face increasing logistical–administrative demands and the widespread use of digital systems. Both may affect job satisfaction and patient safety by increasing workload and contributing to missed nursing care.

**Objective:**

To quantify logistical–administrative workload and satisfaction with health technology among hospital nurses and to identify associations with job satisfaction and the prevalence of missed care.

**Design:**

Cross‐sectional survey.

**Setting(s):**

A national cross‐sectional survey of all nurses working in hospitals in Belgium was conducted between September 16 and October 18, 2024.

**Participants:**

A total of 3613 hospital nurses (79% staff nurses and 21% nurse managers) completed the entire online survey.

**Methods:**

An online questionnaire assessed the proportion of time spent on administrative and logistical tasks, satisfaction with health technologies, job satisfaction, and 15 types of missed nursing care. Multivariable logistic and Poisson regression models, adjusted for sociodemographic and work environment factors, were applied.

**Results:**

Nurses reported spending a median of 18% per shift on logistical–administrative tasks, with staff nurses spending more time than nurse managers (20% vs. 10%, *p* < 0.001) on these tasks. Overall, 71.7% were satisfied with their job, yet 72.1% reported at least one missed care activity. Higher logistical–administrative workload was associated with greater odds of job dissatisfaction (ORa = 1.15, 95% CI [1.10–1.20]) and more missed care (IRRa = 1.12 [1.04–1.20]). Dissatisfaction with health technologies was similarly linked to job dissatisfaction (ORa = 1.30 [1.07–1.58]) and missed care (IRRa = 1.17 [1.10–1.24]). Poor or average work environment was strongly associated with job dissatisfaction (ORa = 15.51 [12.04–19.71]) and missed care (IRRa = 1.36 [1.28–1.50]).

**Conclusions:**

Higher perceived logistical–administrative workload and lower satisfaction with health technologies were associated with increased job dissatisfaction and missed nursing care among hospital nurses. These findings highlight the importance of considering how administrative demands and digital tools are integrated into daily nursing work when addressing working conditions and care quality.

**Highlights**
• What is already known.
◦ Nurses spend considerable time on administrative and digital tasks.
◦ Nurses who are stressed about technology are more likely to feel burned out. However, we still do not know what effect technostress has on patient safety.
◦ Differences between professional roles remain underexplored when it comes to logistical–administrative workload.
• What this paper adds.
◦ Higher logistical–administrative workload and lower satisfaction with digital tools are related to both job dissatisfaction and missed care.
◦ Staff nurses report more missed care, while nurse managers show lower job satisfaction.
• How this study might affect research, practice or policy.
◦ These findings call for a shift from performance‐focused efficiency towards robust practices that sustain nurses and protect patient care.

## 1. Introduction

In recent years, hospital nurses have seen a growing share of their professional time absorbed by logistical–administrative tasks such as documentation, coordination, internal communication and the handling of health information systems. While essential to the proper functioning of care delivery, these tasks are often perceived as burdensome, time‐consuming and distanced from the core of patient care [1]. Observational studies have shown that logistical–administrative duties can occupy up to one‐third of nursing time, raising concerns about the impact of these duties on nurse well‐being, professional satisfaction and care quality [2]. These additional demands add to the overall workload, which has been linked to lower quality of care and reduced job satisfaction [3, 4].

There is now extensive evidence linking job satisfaction among hospital nurses to the quality of their work environment, including leadership support, staffing levels and access to professional resources [5, 6]. Studies conducted in Belgium and across Europe have highlighted the fact that excessive workload and bureaucratic complexity can contribute to burnout and the intention to leave the profession [3, 7]. Nurses increasingly identify the accumulation of administrative duties and poorly designed digital systems as significant barriers to job satisfaction and patient safety [8, 9].

Excessive workload, whether clinical or administrative, has also been repeatedly linked to “missed nursing care,” defined as the partial, delayed or omitted delivery of necessary care [10]. This phenomenon affects not only direct clinical tasks but also essential aspects such as communication with patients and families or the documentation of care [11, 12]. Evidence suggests that missed nursing care is associated with higher risks of adverse patient outcomes, lower perceived quality of care and decreased professional fulfilment among nurses [13]. Addressing the factors that drive missed care is, therefore, critical for both patient safety and workforce sustainability.

From a conceptual standpoint, nursing workload is a multidimensional construct that encompasses direct care, indirect care and non‐patient‐related activities [14]. Some of these non‐nursing tasks are legally required, such as completing the nursing record, while others are primarily organisational in nature. Within this framework, logistical and administrative activities can be viewed as an organisational component of non‐nursing work, as they are driven mainly by coordination processes, material flows, documentation requirements and digital systems rather than by clinical decision‐making. This study deliberately focused on these logistical and administrative activities. This choice is theoretically grounded: this segment of workload is strongly shaped by organisational structures and by how health technologies are introduced and used in daily practice [15]. Logistical tasks, such as supply management or arranging patient transport, are often described as activities that could be delegated to trained support staff, suggesting a potential mismatch between task allocation and nursing qualifications. Administrative tasks are largely determined by documentation demands and digital tools and may increase workload when they are poorly aligned with clinical workflows. Considering these two dimensions together makes it possible to examine a specific form of indirect care workload that is highly sensitive to organisational decisions and to the configuration of care support systems. This perspective is particularly relevant for analysing modifiable aspects of nursing work.

Therefore, what remains less explored is how perceived logistical–administrative workload, together with satisfaction related to health technologies, impacts two key outcomes: job satisfaction and missed nursing care. Although several studies have investigated clinical workload and general work environment factors, few have specifically focused on the subjective burden of these indirect, system‐driven nursing activities and their consequences [5, 16]. Moreover, the experience of logistical–administrative load may vary significantly between professional roles: head nurses, who frequently take on coordination and reporting responsibilities, may perceive and manage this burden differently from bedside nurses [17–19].

Understanding how logistical‐administrative workload affects nursing professionals is essential for improving both staff retention and care delivery. This study aimed to quantify the perceived logistical–administrative workload, assess nurse satisfaction with this workload and with health technology and examine associations between these and overall job satisfaction and the prevalence of missed nursing care among hospital nurses. While this study was primarily exploratory, it drew on growing evidence that logistical–administrative demands weigh heavily on nurses [1, 2] and that dissatisfaction with health technologies undermines care and fulfilment [3, 6, 18]. We, therefore, expected that high‐perceived logistical–administrative workload and limited satisfaction with digital tools would be associated with lower job satisfaction and more missed care.

## 2. Methods

### 2.1. Study Design

This study was a cross‐sectional analysis based on an online survey conducted in Belgium between September 16 and October 18, 2024. The survey was distributed via social media platforms, nursing federations and unions, hospital associations, organizations representing the nursing profession and the Federal Public Health Service. This study followed the STROBE guidelines for the reporting of cross‐sectional studies [20].

### 2.2. Participants

The target population included all actively practicing nurses in Belgium. No employment status or sector of activity was excluded in order to ensure a representative sample of the nursing profession. Nurses who were inactive (e.g., on medical leave or, otherwise, absent) or not practicing in Belgium were excluded. Approximately 15% of the respondents were removed from the dataset due to not meeting the inclusion criteria or submitting incomplete questionnaires and analyses were conducted on complete cases only.

In total, 5535 complete and validated responses were collected from various sectors, including hospitals, nursing homes, home care, and community health. Considering the total number of active nurses in Belgium (154,010 nurses), the sample was deemed sufficiently representative [21]. For the purposes of this study, the analysis was limited to nurses employed in the hospital sector, in order to reduce heterogeneity related to differences in care organisation, workload characteristics and digital tool used across healthcare settings, resulting in a final sample of 3613 respondents. A distinction was made between staff nurses and nurse managers in order to better capture the specific perspectives associated with each role.

### 2.3. Instruments and Measures

This study was conducted using an online questionnaire (LimeSurvey®), and the variables were selected by a working group composed of academics, experts from the Federal Public Health Service and a consulting firm comprising psychologists and sociologists. The instrument included 66 items covering a wide range of topics, including demographic and professional characteristics, activities performed, workload, quality of care, interprofessional collaboration, occupational health and well‐being, working conditions, professional attractiveness and career development.

All survey items included in this study were derived from instruments previously used in Belgian and international nursing workforce research or were developed by a multidisciplinary expert panel. This study included three types of measures: dependent variables, including overall job satisfaction and the prevalence of missed care; independent variables focussing on logistical–administrative workload and control variables to adjust the analyses for the sociodemographic characteristics of nurses, their work environment and hospital‐site characteristics.

Two dependent variables were examined. First, job satisfaction was assessed using a single‐item question in which participants rated their overall satisfaction on a four‐point Likert scale ranging from ‘*very dissatisfied*’ to ‘*very satisfied*.’ This question allowed for the measurement of self‐reported job satisfaction, in line with approaches commonly used in the literature and widely applied in large‐scale nursing workforce studies [22]. Although validated multidimensional instruments have been developed specifically for acute care nurses [23], a single‐item measure was retained here because the questionnaire was part of a large national survey covering multiple domains, where brevity and feasibility were important considerations. Single‐item measures of job satisfaction have demonstrated acceptable validity when the construct is unidimensional and clearly understood by respondents, particularly in large surveys where questionnaire length and respondent burden are key considerations. Second, to assess the prevalence of missed nursing care, nurses were asked to indicate, for 15 specific activities, whether these were necessary but not performed due to time constraints. The selection of these 15 activities is based on the RN4CAST questionnaire, which is widely used internationally to assess unfinished or missed nursing care [24]. The RN4CAST instrument has been implemented in multiple European studies [11, 12]. Its extensive use in hospital‐based research supports the relevance and comparability of the present measure. These activities included items related to clinical care (e.g., adequate patient surveillance, skin care, oral hygiene, pain management, treatments and procedures, timely medication administration, frequent patient repositioning, hand hygiene and airway suctioning), planning and communication (e.g., comforting or discussing with patients or family members, preparing patients for hospital discharge, developing or updating nursing care plans or pathways, care planning and participation in multidisciplinary meetings) and documentation of nursing care [22]. Based on the responses, two indicators were calculated: the number of missed nursing care activities at the individual level and the percentage of nurses reporting each specific missed care activity [13].

Two main independent variables were taken into account. First, logistical–administrative workload was assessed using a self‐administered questionnaire in which nurses were asked to estimate, as a percentage, the average amount of time they spent on various activities during their total working hours over the past 4 weeks. The total had to equal 100%. Administrative and logistical activities were clearly distinguished in the questionnaire. Administrative tasks included entering and updating patient records, care planning, inter team reporting and other documentation‐related activities. Logistical tasks referred to activities such as meal preparation and delivery, processing supply orders and organising or storing equipment and materials. For the analyses, these two categories were combined into a single indicator of logistical–administrative workload, as both refer to organisationally driven, nonclinical activities within indirect care. This theory‐driven aggregation aimed to capture a potentially avoidable component of workload rather than the whole spectrum of indirect care. In addition to these categories, the questionnaire also captured time spent on other core nursing activities, including direct and indirect patient care, family support, participation in multidisciplinary meetings, time dedicated to training, supervision of trainees and other miscellaneous tasks, in order to provide a complete picture of the distribution of working time. For nurse managers, additional categories specific to their coordination role were included, such as managing absences, preparing duty rosters and schedules, coordinating teams and allocating staff. Supporting Table 1 provides a comprehensive overview of all activity categories included in the questionnaire for assessing workload. This questionnaire‐based, self‐reported method has previously been used to comprehensively estimate nurse workload in contexts where standardized workload measurement tools are not available [25]. This approach provides an indicative estimate of workload distribution. The expert‐based development of the instrument supports its content validity although it has not undergone formal psychometric validation as a standalone measure. Second, satisfaction with the use of technological tools provided in the workplace was assessed using a four‐point Likert scale ranging from ‘*very dissatisfied’* to ‘*very satisfied.*’

The control variables encompassed sociodemographic characteristics, work environment factors and hospital‐related attributes. Specifically, these included gender, professional experience, region of employment, educational qualification, marital status, nationality, patient‐to‐nurse ratio, care setting, hospital unit, hospital type and job position. The work environment was assessed using a single item overall measure (‘How would you rate your working environment as a whole?’) offering four response options ranging from ‘*very poor*’ to ‘*excellent*.’ This type of global assessment provides a concise indicator that has been used in large‐scale nursing workforce surveys to reduce respondent burden and ensure feasibility within comprehensive national questionnaires [26]. These variables were selected in accordance with the literature, as they are known to influence job satisfaction, and prevalence of missing care [3, 22, 27].

### 2.4. Statistical Analyses

Descriptive statistics were used to summarize the data. For quantitative variables, the median and interquartile range (IQR) are reported, while absolute frequencies (*n*) and relative frequencies (%) are used for qualitative variables. The Kruskal–Wallis test was applied to compare quantitative variables across groups, and the chi‐squared (χ^2^) test was used for comparisons involving qualitative variables. Nurses are considered key sources of information for assessing healthcare performance at the individual level. Two separate multivariable regression models were conducted.

First, a logistic regression tested the association between the independent variables—logistical–administrative workload (categorized into tertiles: low, medium and high) and satisfaction with the use of technological tools (dichotomised as very/satisfied vs. very/dissatisfied)—and job dissatisfaction. Results are presented as adjusted odds ratios (ORas) with 95% confidence intervals (CIs). Second, a zero‐inflated Poisson (ZIP) regression model was used to assess the association between the same independent variables and the number of missed nursing care activities. A Vuong likelihood‐ratio test was conducted to confirm that the ZIP model provided a superior fit compared to standard Poisson models, justifying its use given the excess zero counts observed in the outcome distribution (Vuong *z* = 3.21, *p* = 0.0013). Results are presented as adjusted incidence rate ratios (IRRas), interpreted similarly to odds ratios (IRR > 1.0 indicating a higher frequency of missed care), with 95% CIs and *p* values. Model quality was assessed using the Akaike Information Criterion (AIC). Model selection was informed by AIC values obtained during the modelling process. Both models were adjusted for control variables, including sociodemographic characteristics, work environment factors and hospital‐related attributes (e.g., gender, professional experience, region of employment, educational qualification, marital status, nationality, patient‐to‐nurse ratio, care setting, hospital unit, hospital type and job position). All regression analyses were performed using a generalized estimating equations (GEE) approach with an independence correlation matrix to account for potential clustering. Statistical significance was set at *p* < 0.05. Analyses were conducted using STATA Version 18 and R Version 4.3.2.

### 2.5. Ethical Considerations

All participants provided informed consent prior to participation. They were clearly informed about the voluntary nature of their involvement, as well as the anonymity and confidentiality of the data collected. The study received ethical approval from the Ethics Committee of CHU de Liège, Belgium (approval no. 2025/583). The study was conducted in accordance with the ethical principles outlined in the Declaration of Helsinki and complies with the General Data Protection Regulation (GDPR, EU 2016/679). The data are used solely for scientific research purposes.

## 3. Results

### 3.1. Description of Nurse Characteristics

A total of 3613 nurses were included in this study. Most respondents were women (81.6%), worked as frontline nursing staff (79.0%) and held either a bachelor’s degree or a specialization in nursing (70.2%). The median age was 43 years [35–53], median professional experience was 19 years [10–30] and 56.6% worked full time. Nearly half of the sample (45.8%) had completed a postbasic specialization. The median patient‐to‐nurse ratio was 9 [3–20], and perceptions of the work environment were almost evenly distributed between ‘poor or average’ (48.4%) and ‘good or excellent’ (51.6%). Nurses from all hospital unit types were represented, with the largest groups working in general medical–surgical wards (33.7%) and ICU/ED/Neonatology units (24.2%) (Table 1).

**TABLE 1:** Description of nurse characteristics.

Variables	Results (*n* = 3613)
Age (year), median [p25–p75]	43 [35–53]
Gender, female, *n* (%)	2949 (81.6)
Experience (years), median [p25–p75]	19 [10–30]
Full‐time employed, *n* (%)	2045 (56.6)
Region, *n* (%)	
Flanders	1288 (35.6)
Brussels	849 (23.5)
Wallonia	1476 (40.9)
Diploma, *n* (%)	
No bachelor’s degree	545 (15.1)
Bachelor’s degree	880 (24.4)
Specialized nurses	1654 (45.8)
University degree	524 (14.5)
Doctorate	10 (0.3)
Marital status, *n* (%)	
Single without children	618 (17.1)
Single with children	359 (9.9)
Cohabiting/married without children	605 (16.8)
Cohabiting/married with children	2031 (56.2)
Patient‐to‐nurse ratio, median [p25–p75]	9 [3–20]
Nationality, *n* (%)	
Belgian	3314 (91.7)
EU 27	256 (7.1)
Outside EU 27	43 (1.2)
Working environment, *n* (%)	
Poor or average	1749 (48.4)
Good or excellent	1864 (51.6)
Units, *n* (%)	
Nursing department management	49 (1.4)
ICU/ED/Neonatology	912 (24.2)
Ward general wards	1219 (33.7)
Medico‐technical units	728 (20.1)
Mobile team	539 (14.9)
Others	166 (4.6)
Type of working hospital, *n* (%)	
General hospital	2451 (67.8)
Academic hospital	1162 (32.2)
Position, *n* (%)	
Staff nurse	2856 (79.0)
Nurse manager	757 (21.0)

Abbreviations: ED: emergency department; EU: European Union; ICU: intensive care unit.

### 3.2. Description of Job Satisfaction Work and Prevalence of Missed Nursing Care

**FIGURE 1:**
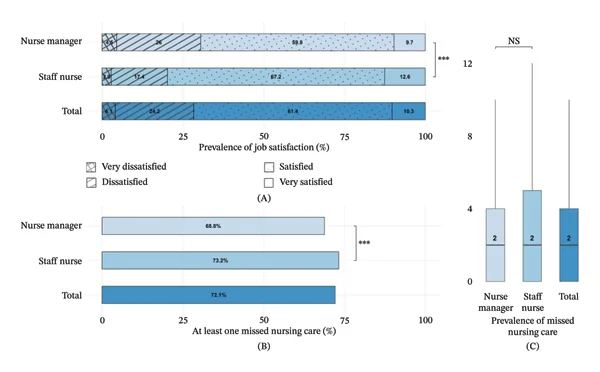
Prevalence of satisfaction job (A) and missed nursing care (B and C) stratified by position. Significance levels: ^∗^ *p* value < 0.05, ^∗∗^ *p* value < 0.01, ^∗∗∗^ *p* value < 0.001, NS: nonsignificant.

Overall, 71.7% of the nurses reported being satisfied (61.4%) or very satisfied (10.3%) with their job, while 28.3% reported being dissatisfied (24.2%) or very dissatisfied (4.1%). Job satisfaction was significantly lower among nurse managers than among staff nurses (69.5% vs. 79.8%; *p* < 0.001). Regarding missed nursing care, 72.1% of the respondents reported having omitted at least one activity. This proportion was slightly lower among nurse managers than among staff nurses (68.8% vs. 73.2%; *p* < 0.001). However, the median number of missed care activities was identical in both groups, at two activities, and no significant difference was observed in the total number of missed care activities (Figure 1).

### 3.3. Logistical–Administrative Workload and Satisfaction With Health Technologies

**FIGURE 2:**
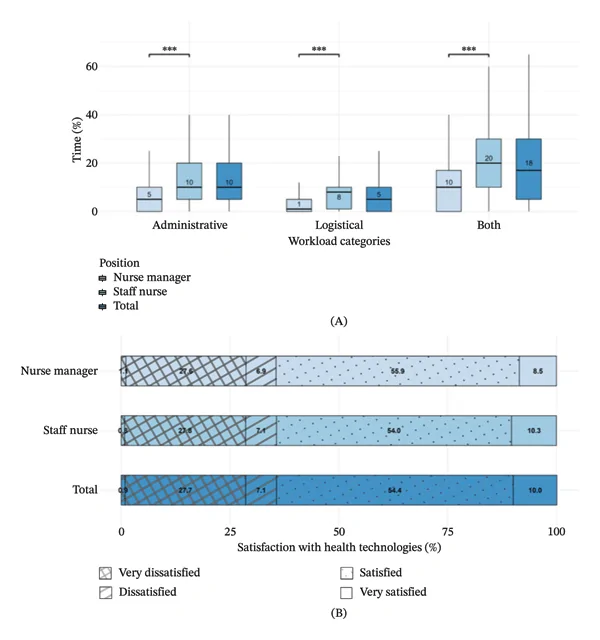
Logistical–administrative workload and satisfaction with health technologies stratified by position. Significance levels: ^∗^ *p* value < 0.05, ^∗∗^ *p* value < 0.01, ^∗∗∗^ *p* value < 0.001, NS: non‐significant.

Staff nurses reported spending a significantly greater proportion of their time on logistical‐administrative tasks than nurse managers. Overall, the median time dedicated to these tasks was 18% per shift [IQR: 5–30], including 5 [0–10] for logistical activities and 10 [5–20] for administrative tasks. When stratified by hierarchical position, nurse managers reported 10% [0–18] of their time spent on these tasks, compared with 20% [10–30] among staff nurses (*p* < 0.001). Overall, 64.4% of the respondents reported being satisfied or very satisfied with health technologies, whereas 35.7% reported being dissatisfied or very dissatisfied. This distribution was similar between nurse managers and staff nurses (Figure 2).

Across workload categories, direct care and indirect care accounted for the largest share of nurses’ working time. Both differed significantly according to hierarchical position (*p* < 0.001), with nurse managers reporting substantially less time spent on direct care than staff nurses (5.0% [0–15] vs. 35.0% [25–50]) (Supporting Table 1).

### 3.4. Multivariate Association Between Logistical–Administrative Workload, Satisfaction With Health Technology and Dependent Variables

**FIGURE 3:**
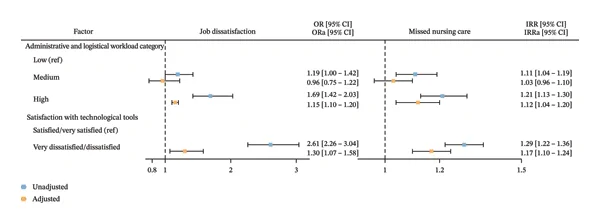
Multivariate association between logistical–administrative workload, satisfaction with health technologies and dependent variables, including job satisfaction and prevalence of missed care. Significance levels: ^∗^ *p* value < 0.05, ^∗∗^ *p* value < 0.01, ^∗∗∗^ *p* value < 0.001; ORa: odds ratio adjusted; IRRa: adjusted incident rate ratio; REF: reference; control variables (gender, position, nurse education, experience, marital status, working environment, working hours, patient‐to‐nurse ratio, nationality, units, type of hospital and position) are included in each multivariate model but not reported in the tables (see supporting Figure 1). Akaike Information Criterion (AIC) values for the models: job dissatisfaction (AIC = 2737) and missed nursing care (AIC = 13,012).

In the multivariate analysis, a higher logistical–administrative workload was significantly associated with 15% higher odds of job dissatisfaction (ORa = 1.15, 95% CI: [1.10–1.20]) and a 12% higher frequency of missed nursing care (IRRa = 1.12 [1.04–1.20]). Similarly, nurses who reported dissatisfaction with technological tools had a 30% higher risk of job dissatisfaction (ORa = 1.30 [1.07–1.58]) and a 17% higher prevalence of missed care (IRRa = 1.17 [1.10–1.24]) (Figure 3).

Among the covariates, a poor or average work environment was strongly associated with both job dissatisfaction (ORa = 15.51 [12.04–19.71]) and missed nursing care (IRRa = 1.36 [1.28–1.50]). Male gender was also associated with higher odds of job dissatisfaction (ORa = 1.41 [1.11–1.82]) and a higher frequency of missed nursing care (IRRa = 1.11 [1.04–1.19]). By contrast, nurse manager position was not significantly associated with either job dissatisfaction (ORa = 0.78 [0.58–1.06]) or missed nursing care (IRRa = 1.02 [0.94–1.16]). Compared with Flanders, nurses working in Brussels and Wallonia reported higher odds of job dissatisfaction and slightly higher frequencies of missed nursing care. The full set of covariates is presented in Figure 3, with additional details in Supporting Figure 1.

## 4. Discussion

This study examined logistical–administrative workload and satisfaction with health technologies among hospital nurses and their associations with job satisfaction and missed nursing care. As an exploratory study, it identified consistent associations that help clarify how nonclinical demands and digital environments relate to nursing work and care delivery.

Our first objective was to quantify perceived logistical–administrative workload. This workload appeared to represent a substantial share of working time and to vary according to hierarchical position. Staff nurses reported spending more time on these activities while remaining more involved in direct and indirect care, a pattern that may be related to role definitions rather than to an unequal distribution of tasks. Frontline roles tend to combine clinical care with documentation, care planning and patient‐flow coordination in daily patient management [1, 14], suggesting that logistical–administrative activities could be understood as part of indirect care structurally embedded in bedside practice [1, 14]. Nurse managers reported lower exposure to these activities and greater involvement in coordination, planning and oversight. This configuration is consistent with organisational models in which managerial roles are more oriented toward system‐level responsibilities, whereas frontline roles integrate both clinical and operational work [22]. Overall, these results may indicate role‐specific configurations of logistical–administrative workload. Interpreting them in relation to professional functions may help to better understand how this workload is experienced in daily practice, particularly in contexts of sustained clinical and organisational pressure [27, 28].

Our second objective was to explore the relationship between nurses’ satisfaction with health technologies and perceived logistical–administrative workload. Lower satisfaction was associated with a higher perceived workload across roles although staff nurses reported slightly lower satisfaction than nurse managers. The survey did not include qualitative data, so the reasons for this dissatisfaction cannot be identified, and no conclusion can be drawn as to whether it relates to the tools themselves or to other factors. When compared with data collected in 2019 in a similar hospital nursing population, the proportion of nurses reporting dissatisfaction appears to have increased, which may reflect a context marked by the rapid expansion of digital systems in recent years [15]. Previous studies suggest that perceived digital burden may be linked to usability, workflow integration and cumulative documentation demands rather than to the mere presence of technology [29, 30]. The observed difference between staff nurses and nurse managers may be interpreted in light of differences in exposure. Frontline nurses are more frequently and directly engaged with documentation and data‐entry systems during clinical activity, which may shape their experience of digital tools more strongly than that of managers [3, 30]. This interpretation remains hypothetical but is consistent with studies describing technostress as a context‐dependent phenomenon associated with task intensity and time pressure [31]. Limited involvement of end users in the design, testing and implementation of digital tools has also been reported as a potential source of dissatisfaction [6, 29]. Taken together, these elements suggest that satisfaction with health technologies may depend not only on technical performance but also on how tools are selected, implemented and integrated into daily clinical work. Further qualitative or mixed‐methods studies would be needed to explore these mechanisms.

Our third objective was to examine whether higher logistical–administrative workload and lower satisfaction with health technologies were associated with job dissatisfaction and missed nursing care. Adjusted analyses suggest that both higher perceived workload and lower satisfaction with digital tools were associated with a greater likelihood of job dissatisfaction and a higher frequency of missed nursing care. As job satisfaction was measured as a global perceived outcome, these associations should be interpreted as reflecting overall professional satisfaction rather than its specific dimensions and remain correlational and hypothesis‐generating. This pattern is consistent with studies showing that missed nursing care is mainly related to time pressure, competing demands and task fragmentation rather than individual performance, suggesting that administrative and digital demands may reduce the time and cognitive resources available for care [32, 33]. Regional differences were also observed, with higher job dissatisfaction and slightly more missed nursing care reported in Wallonia and Brussels than in Flanders. Similar patterns have been described in previous Belgian studies, where variation in work environment characteristics was associated with differences in job satisfaction and retention [7, 22, 34, 35]. One possible explanation relates to broader cultural and organisational contexts: the Flemish healthcare system is often described as closer to an Anglo‐Saxon model with earlier investment in quality improvement and nursing work environments, whereas Wallonia and parts of Brussels are more influenced by a Latin organisational tradition where hierarchical structures and resource constraints may shape professional experience differently [36]. This interpretation remains contextual and noncausal.

### 4.1. Practical Implications for Healthcare Settings

Taken together, our findings suggest that the associations between workload, digital tools, job dissatisfaction and missed care cannot be addressed by focussing on interventions alone. Digital and organisational changes may offer benefits, but their effects are likely to depend on how they are implemented and integrated into daily practice [37]. In this regard, implementation science offers a relevant framework to understand why some interventions do not produce the expected effects and to shift attention from the tools themselves to the processes surrounding their adoption, including user involvement, contextual adaptation and continuous feedback. Future research could extend these findings through unit‐level analyses, as workload configuration, digital tool use and care processes are likely to differ across clinical contexts. Our results also invite caution regarding narrowly optimisation‐driven approaches to nursing work, as an exclusive focus on efficiency may overlook care complexity and the long‐term sustainability of the workforce.

### 4.2. Limitations and Strengths

This study has several limitations. The cross‐sectional design prevents establishing causal relationships between workload, job satisfaction and missed nursing care. Also, the voluntary nature of participation may have introduced selection bias, as hospitals or nurses more concerned about working conditions may have been more likely to respond. Data were self‐reported, which may have led to reporting bias related to subjective perceptions, recall or social desirability. Workload was assessed using a self‐administered questionnaire in which nurses allocated percentages of their working time across activities. While this approach provides a broad overview of workload distribution and has been used in previous studies, it remains an indirect and subjective measure that may not fully capture the complexity and variability of nursing tasks across shifts, units or patient acuity levels and should, therefore, be interpreted as an indicative estimate of workload distribution. Although the instrument was developed through an expert‐driven process supporting its content validity, it has not undergone formal psychometric validation as a standalone measure. Future work could build on methodological approaches used in the psychometric evaluation of nursing workforce instruments (e.g., Reference [23]). In addition, the theory‐driven aggregation of administrative and logistical activities into a single indicator may have obscured specific organisational mechanisms underlying each component. In addition, analyses were conducted at the hospital level and no subgroup analyses by unit type were performed. Given the heterogeneity of workload organisation across clinical settings (e.g., intensive care, emergency and medical–surgical units), the findings should not be directly generalised to all hospital units. Although key control variables identified in the literature were included in the models, residual confounding cannot be ruled out, as some organisational factors (e.g., staffing models, skill mix, IT support availability or patient acuity) may have influenced the observed associations. The lack of longitudinal data further limits the assessment of changes over time or intervention effects. Despite these limitations, this study has important strengths. The large and diverse national sample enhances the generalisability of the findings within Belgian hospital settings. The questionnaire was developed through collaboration between academic, public health and social science experts, ensuring relevance and content validity. Finally, the use of GEE allowed for robust multivariable analyses accounting for clustering at the hospital level. Future research should build on these findings using longitudinal designs and more objective workload measurement approaches.

## 5. Conclusions

This study shows that logistical–administrative workload and satisfaction with digital tools are associated with nurses’ job satisfaction and missed nursing care. Nurses reporting higher nonclinical workload and lower satisfaction with health technologies were more likely to report job dissatisfaction and omissions in care. These findings underline the importance of considering how logistical–administrative tasks and digital tools are embedded in daily nursing work when addressing working conditions and care quality.

## Author Contributions

MP: methodology, conceptualisation and writing–original draft; GD: writing–review and editing; JD: formal analysis and review and editing; AB: methodology, supervision, formal analysis, conceptualisation and writing–review and editing.

## Funding

No funding was received for this research.

## Disclosure

All authors have approved the final version of the report.

## Ethics Statement

All participants provided informed consent prior to participation. They were clearly informed about the voluntary nature of their involvement, as well as the anonymity and confidentiality of the data collected. The study received ethical approval from the Ethics Committee of CHU de Liège, Belgium (approval no. 2025/583). The study was conducted in accordance with the ethical principles outlined in the Declaration of Helsinki and complies with the General Data Protection Regulation (GDPR, EU 2016/679). The data are used solely for scientific research purposes.

## Consent

The authors affirm that signed informed consent was obtained from all individual participants included in this study.

## Conflicts of Interest

The authors declare no conflicts of interest.

## Supporting Information

Additional supporting information can be found online in the Supporting Information section.

## Supporting information


**Supporting Information 1** Supporting Table 1. Detailed breakdown of workload distribution by hierarchical position (nurse managers vs. staff nurses). In addition to the categories shown in Figure 2, this table reports other components of nurses’ working time, allowing a more comprehensive overview of how tasks are allocated.

**Supporting Information 2** Supporting Figure 1. Complete set of covariates included in the adjusted multivariate models presented in Figure 3. This figure provides additional details on the control variables used, clarifying their contribution to the associations between workload, satisfaction with health technology, job dissatisfaction and missed nursing care.

## Data Availability

The dataset used and analysed in this manuscript are available from the corresponding author upon reasonable request.

## References

[1] Michel L. , A Failure to Communicate? Doctors and Nurses in American Hospitals, Journal of Health Politics Policy and Law. (2017) 42, no. 4, 709–717, 10.1215/03616878-3856149. 28483809

[2] Ganty T. , Szecel J. , Diep A. , Ghuysen A. , and Paquay M. , Multicentre Cross-Sectional Study to Assess Nursing Workload in Belgian Emergency Departments, Emergency Medicine Journal. (2025) 42, no. 7, 421–428, 10.1136/emermed-2024-214334. 39947875 PMC12229054

[3] Aiken L. H. , Sermeus W. , McKee M. et al., Physician and Nurse Well-Being, Patient Safety and Recommendations for Interventions: Cross-Sectional Survey in Hospitals in Six European Countries, BMJ Open. (2024) 14, no. 2, 10.1136/BMJOPEN-2023-079931. PMC10862305 38346890

[4] Bruyneel A. , Dello S. , Dauvergne J. E. , Kohnen D. , and Sermeus W. , Prevalence and Risk Factors for Burnout, Missed Nursing Care, and Intention-to-Leave the Job Among Intensive Care Unit and General Ward Nurses: A Cross-Sectional Study Across Six European Countries in the COVID-19 Era, Intensive and Critical Care Nursing. (2025) 86, 10.1016/j.iccn.2024.103885. 39522308

[5] Kohnen D. , De Witte H. , Schaufeli W. B. , Dello S. , Bruyneel L. , and Sermeus W. , Engaging Leadership and Nurse Well-Being: The Role of the Work Environment and Work Motivation—A Cross-Sectional Study, Human Resources for Health. (2024) 22, no. 1, 10.1186/s12960-023-00886-6. PMC10788988 38225620

[6] Zhao Y. , Lu H. , Zhu X. , and Xiao G. , Job Satisfaction Among Hospital Nurses: An Updated Literature Review, International Journal of Nursing Studies. (2025) 162, 10.1016/j.ijnurstu.2024.104964. 39642613

[7] Bruyneel A. , Bouckaert N. , de Noordhout C. M. et al., Association of Burnout and Intention-to-Leave the Profession With Work Environment: A Nationwide Cross-Sectional Study Among Belgian Intensive Care Nurses After Two Years of Pandemic, International Journal of Nursing Studies. (2022) 137, 10.1016/j.ijnurstu.2022.104385. PMC9640385 36423423

[8] Borges do Nascimento I. J. , Abdulazeem H. , Vasanthan L. T. et al., Barriers and Facilitators to Utilizing Digital Health Technologies by Healthcare Professionals, NPJ Digital Medicine. (2023) 6, no. 1, 10.1038/s41746-023-00899-4. PMC10507089 37723240

[9] Beks H. , Clayden S. , Wong Shee A. et al., Low-Value Health Care, De-Implementation, and Implications for Nursing Research: A Discussion Paper, International Journal of Nursing Studies. (2024) 156, 10.1016/j.ijnurstu.2024.104780. 38744150

[10] Jones T. L. , Hamilton P. , and Murry N. , Unfinished Nursing Care, Missed Care, and Implicitly Rationed Care: State of the Science Review, International Journal of Nursing Studies. (2015) 52, no. 6, 1121–1137, 10.1016/j.ijnurstu.2015.02.012. 25794946

[11] Ball J. E. , Bruyneel L. , Aiken L. H. et al., Post-Operative Mortality, Missed Care and Nurse Staffing in Nine Countries: A Cross-Sectional Study, International Journal of Nursing Studies. (2018) 78, 10–15, 10.1016/j.ijnurstu.2017.08.004. 28844649 PMC5826775

[12] Griffiths P. , Recio-Saucedo A. , Dall’Ora C. et al., The Association Between Nurse Staffing and Omissions in Nursing Care: A Systematic Review, Journal of Advanced Nursing. (2018) 74, no. 7, 1474–1487, 10.1111/jan.13564. 29517813 PMC6033178

[13] Bruyneel A. , Bouckaert N. , Pirson M. , Sermeus W. , and Van den Heede K. , Unfinished Nursing Care in Intensive Care Units and the Mediating Role of the Association Between Nurse Working Environment, and Quality of Care and Nurses’ Wellbeing, Intensive and Critical Care Nursing. (2024) 81, 10.1016/j.iccn.2023.103596. 38043435

[14] Alghamdi M. G. , Nursing Workload: A Concept Analysis, Journal of Nursing Management. (2016) 24, no. 4, 449–457, 10.1111/jonm.12354. 26749124

[15] Van Den Heede K. , Bruyneel L. , Beeckmans D. et al., Safe Nurse Staffing Levels in Acute Hospitals, 2019, Belgian Health Care Knowledge Centre (KCE), 10.57598/R325C.

[16] Yang J. , Amrollahi A. , and Marrone M. , Harnessing the Potential of Artificial Intelligence: Affordances, Constraints, and Strategic Implications for Professional Services, Journal of Strategic Information Systems. (2024) 33, no. 4, 10.1016/j.jsis.2024.101864.

[17] Laukka E. , Hammarén M. , Pölkki T. , and Kanste O. , Hospital Nurse Leaders’ Experiences With Digital Technologies: A Qualitative Descriptive Study, Journal of Advanced Nursing. (2023) 79, no. 1, 297–308, 10.1111/jan.15481. 36300725 PMC10092210

[18] Laakkonen N. , Jarva E. , Hammarén M. et al., Digital Competence Among Healthcare Leaders: A Mixed-Methods Systematic Review, Journal of Nursing Management. (2024) 2024, no. 1, 10.1155/2024/8435248. PMC11919023 40224897

[19] Navarro Martínez O. and Leyva-Moral J. M. , Digital Transformation Led by Nurses and Nursing Managers’ Priorities: A Qualitative Study, Journal of Nursing Management. (2024) 2024, no. 1, 10.1155/2024/8873127. PMC11918896 40224899

[20] von Elm E. , Altman D. G. , Egger M. , Pocock S. J. , Gøtzsche P. C. , and Vandenbroucke J. P. , The Strengthening the Reporting of Observational Studies in Epidemiology (STROBE) Statement: Guidelines for Reporting Observational Studies, Lancet. (2007) 370, no. 9596, 1453–1457, 10.1016/S0140-6736(07)61602-X. 18064739

[21] Vivet V. , Durand C. , Jouck P. , Nkenne D. , and Steinberg P. , Infirmiers Sur Le Marché Du Travail, 2024, https://organesdeconcertation.sante.belgique.be/fr/documents/infirmiers-sur-le-marche-du-travail-2022.

[22] Sermeus W. , Aiken L. H. , den Heede K. V. et al., Nurse Forecasting in Europe (RN4CAST): Rationale, Design and Methodology, BMC Nursing. (2011) 10, no. 1, 10.1186/1472-6955-10-6. PMC3108324 21501487

[23] Yasin Y. M. , Kehyayan V. , Khraim F. , and Al-Lenjawi B. , Psychometric Evaluation of the Acute Care Nurses’ Job Satisfaction Scale-Revised, Nursing Open. (2022) 10, no. 1, 488–497, 10.1002/nop2.1314. 36054793 PMC9834183

[24] Ausserhofer D. , Zander B. , Busse R. , Schubert M. , De Geest S. , Rafferty A. M. , Ball J. , Scott A. , Kinnunen J. , Heinen M. , Strømseng Sjetne I. , Moreno-Casbas T. , Kózka M. , Lindqvist R. , Diomidous M. , Bruyneel L. , Sermeus W. , Aiken L. H. , and Schwendimann R. , Prevalence, Patterns and Predictors of Nursing Care Left Undone in European Hospitals: Results From the Multicountry Cross-Sectional RN4CAST Study, BMJ Quality and Safety. (2014) 23, no. 2, 126–135, 10.1136/bmjqs-2013-002318. 24214796

[25] Racy S. , Davidson P. M. , Peeler A. , Hager D. N. , Street L. , and Koirala B. , A Review of Inpatient Nursing Workload Measures, Journal of Clinical Nursing. (2021) 30, no. 13-14, 1799–1809, 10.1111/jocn.15676. 33503306

[26] Lake E. T. , Gil J. , Moronski L. , McHugh M. D. , Aiken L. H. , and Lasater K. B. , Validation of a Short Form of the Practice Environment Scale of the Nursing Work Index: The PES-5, Research in Nursing & Health. (2024) 47, no. 4, 450–459, 10.1002/nur.22388. 38669131 PMC11236491

[27] Caillet A. , Dauvergne J. E. , Poiroux L. , Blanchard P. Y. , and Bruyneel A. , Analysis of the Prevalence of Missed Nursing Care Using Three Workload Assessment Methods: A Nationwide Cross-Sectional Study Among Intensive Care Nurses, Nursing in Critical Care. (2025) 30, no. 3, 10.1111/nicc.70055. 40384582

[28] Kruk M. E. , Gage A. D. , Arsenault C. et al., High-Quality Health Systems in the Sustainable Development Goals Era: Time for a Revolution, Lancet Global Health. (2018) 6, no. 11, e1196–e1252, 10.1016/S2214-109X(18)30386-3. 30196093 PMC7734391

[29] Keshavarz H. , Saeidnia H. R. , and Wang T. , Navigating Technostress: A Deep Dive Into Health Practitioners’ Technological Challenges in Hospital Settings, BMC Health Services Research. (2025) 25, no. 1, 10.1186/s12913-024-12196-1. PMC11699643 39754088

[30] Kopuz K. , Turgut M. , and Aydın G. , Technostress Among Nurses: Boon or Bane? The Moderated Mediation Model, BMC Nursing. (2025) 24, no. 1, 10.1186/s12912-025-03721-6. PMC12366391 40836237

[31] Asgari E. , Kaur J. , Nuredini G. et al., Impact of Electronic Health Record Use on Cognitive Load and Burnout Among Clinicians: Narrative Review, JMIR Medical Informatics. (2024) 12, 10.2196/55499. PMC11053390 38607672

[32] Papathanasiou I. , Tzenetidis V. , Tsaras K. , Zyga S. , and Malliarou M. , Missed Nursing Care; Prioritizing the Patient’s Needs: An Umbrella Review, Healthcare. (2024) 12, no. 2, 10.3390/healthcare12020224. PMC10815730 38255111

[33] Wirth T. , Kräft J. , Marquardt B. , Harth V. , and Mache S. , Indicators of Technostress, Their Association With Burnout and the Moderating Role of Support Offers Among Nurses in German Hospitals: A Cross-Sectional Study, BMJ Open. (2024) 14, no. 7, 10.1136/bmjopen-2024-085705. PMC11253736 39002964

[34] Van den Heede K. , Florquin M. , Bruyneel L. et al., Effective Strategies for Nurse Retention in Acute Hospitals: A Mixed Method Study, International Journal of Nursing Studies. (2013) 50, no. 2, 185–194, 10.1016/j.ijnurstu.2011.12.001. 22204812

[35] Van den Heede K. , Bouckaert N. , Detollenaere J. et al., Nurse Staffing on Belgian Intensive Care Units: The Impact of Two Years of COVID-19 Pandemic, 2022, Belgian Healthcare Knowledge Centre (KCE), https://www.kce.fgov.be, Accessed July 4, 2022.

[36] Sermeus W. , Aiken L. H. , Ball J. et al., A Workplace Organisational Intervention to Improve Hospital Nurses’ and Physicians’ Mental Health: Study Protocol for the Magnet4Europe Wait List Cluster Randomised Controlled Trial, BMJ Open. (2022) 12, no. 7, 10.1136/bmjopen-2021-059159. PMC9341186 35902190

[37] Paquay M. , Simon R. , Ancion A. , Graas G. , and Ghuysen A. , A Success Story of Clinical Debriefings: Lessons Learned to Promote Impact and Sustainability, Frontiers in Public Health. (2023) 11, 10.3389/fpubh.2023.1188594. PMC10354544 37475771

