# Supplementary material for: The Work Behind the Work: Logistical–Administrative Workload, Health Technology Satisfaction and Missed Nursing Care Among Belgian Hospital Nurses
Source: J Nurs Manag. 2026 Aug 30;2026:4981208. doi: 10.1155/jonm/4981208 (PMC13526235; doi:10.1155/jonm/4981208)
Supplement: Supplementary file 1 [file JONM-2026-4981208-s001.docx]

**Supplementary Figure 1.** Control variables included in each multivariate model presented in **Figure 3**

Legend: Significance levels: * p-value < 0.05, ** p-value < 0.01, *** p-value < 0.001; ORa: Odds ratio adjusted; IRRa: adjusted incident rate ratio
