# Supplementary material for: The Work Behind the Work: Logistical–Administrative Workload, Health Technology Satisfaction and Missed Nursing Care Among Belgian Hospital Nurses
Source: J Nurs Manag. 2026 Aug 30;2026:4981208. doi: 10.1155/jonm/4981208 (PMC13526235; doi:10.1155/jonm/4981208)
Supplement: Supplementary file 2 [file JONM-2026-4981208-s002.docx]

**Supplementary Table 1:** Other categories of workload by position in addition to **Figure 2**

| **Workload category** | **Nurse Manager**  Median [IQR] | **Staff nurse** Median [IQR] | **pvalue** | **Total** Median [IQR] |
| --- | --- | --- | --- | --- |
| Direct care | 5 [0-15] | 35 [25-50] | < 0.001 | 30 [15-50] |
| Indirect care | 5 [0-10] | 15 [10-20] | < 0.001 | 10 [5-20] |
| Family | 4 [0-10] | 5 [2-10] | < 0.001 | 5 [1-20] |
| Multi-disciplinary concertation | 5 [0-10] | 5 [0-10] | < 0.001 | 5 [0-10] |
| Continuing education | 0 [0-5] | 0 [0-2] | < 0.001 | 0 [0-4] |
| Support for students | 0 [0-5] | 0 [0-5] | 0.005 | 0 [0-5] |
| Team coordination | 15 [5-25] | - | - | - |
| Timetable management | 10 [1-20] | - | - | - |
| Development of procedures | 1 [0-6] | - | - | - |
| Absence management | 10 [2-20] | - | - | - |
| Others | 0 [0-5] | 0 [0-3] | 0.001 | 0 [0-3] |

Legend: IQR: interquartile range.
